# Characteristics of state waivers to establish nutritional restrictions in the supplemental nutrition assistance program

**DOI:** 10.1093/haschl/qxaf221

**Published:** 2025-11-19

**Authors:** Benjamin W Chrisinger

**Affiliations:** Department of Community Health, Tufts University, Medford, MA 02155, United States; Center for Health and Wellbeing, Princeton University, Princeton, NJ 08544, United States

**Keywords:** Supplemental Nutrition Assistance Program, food assistance, sugar-sweetened beverages, state policy, nutrition policy

## Abstract

**Introduction:**

States have previously requested permission from the US Department of Agricultureto implement food item restrictions within the Supplemental Nutrition Assistance Program (SNAP), though these proposals were previously rejected. The current administration is encouraging states to submit waivers for restriction and has approved 12 states to restrict SNAP starting in January 2026.

**Methods:**

This study analyzed states’ waiver proposals and approval letters to describe the landscape of forthcoming restrictions, their justifications and proposed evaluations. Framework analysis was used to identify common terms and themes between states.

**Results:**

Soda restrictions were common across all approved waiver states, with 8 states also implementing some form of restriction on candy. As justification for restriction, states cited the “intended purpose of SNAP” for nutrition (*n* = 9), Medicaid and healthcare costs (*n* = 8), promoting healthy eating (*n* = 8), stewardship of taxpayer dollars (*n* = 7) and high SNAP spending on unhealthy goods (*n* = 6). Evaluations using mixed-methods and pre-post analyses were most common (*n* = 7), with nearly all states proposing the use of retailer data and participant surveys. Key outcomes included purchasing patterns (*n* = 10), dietary patterns (*n* = 5) and health or disease outcomes (*n* = 5).

**Conclusion:**

Substantial variation in restrictions and evaluation approaches warrants attention by policymakers and researchers.

Key Takeaways

- Twelve states have been approved to begin applying nutritionally-informed restrictions to the Supplemental Nutrition Assistance Program.
- Definitions of restricted items and exemptions vary widely between states, though soda was the most restricted item.
- Future impact evaluations should note differences in item definitions and implementation plans between states.

## Introduction

The Supplemental Nutrition Assistance Program (SNAP) serves 41.7 million individuals and has been shown to be an effective safeguard against food insecurity.^1-3^ Participants redeem these benefits at approved food retailers, with relatively few restrictions on the food items purchased. Over the last 15 years, states have debated and requested restrictions from the US Department of Agriculture (USDA) to implement additional food item restrictions, including those with nutrition-focused aims.^4^ Previously, these proposals were uniformly rejected, with USDA citing administrative burden on retailers and participant impacts.^5^ The current Administration has signaled an interest in SNAP restrictions as part of its “Make America Healthy Again” (MAHA) agenda. As of September 2025, USDA has approved item restriction proposals of 12 states, with implementation to begin as early as January 2026.

In all states, SNAP benefits cannot be used to purchase tobacco, products containing controlled substances (eg, cannabis, CBD), vitamins or supplements, live animals, prepared hot foods, or non-food items.^6^ To implement additional SNAP restrictions, states must apply for a waiver of normal SNAP administration procedures, following a template provided by the US Food and Nutrition Service (FNS).^7^ Proposals must include a justification for the request and description of how the waiver will be implemented and evaluated. If approved, FNS provides a formal letter outlining the terms of waiver, including details of the restriction, as well as proposed evaluation procedures and a timeline for quarterly reporting. Approval letters also outline expectations around program administration, such as the need for continued inter-operability between states (eg, participants must be able to use SNAP benefits across state lines). Waivers are granted for an initial period of 2 years, with the option to request annual extensions for 3 additional years.

These forthcoming SNAP restrictions are a substantial change to the modern program, and are, in part, motivated by concerns over diet quality and diet-related non-communicable diseases among SNAP participants.^8^ It is likely that more states will pursue restriction waivers, especially considering a recent announcement that states’ implementation of so-called MAHA policies, like SNAP restriction waivers, will be used to calculate state funding under the federal Rural Health Transformation Program, a fund created to partially offset reduced federal Medicaid spending.^9^ State evaluations of SNAP restrictions may offer evidence on their health impacts, which have long been debated because of inconclusive evidence and possible adverse effects for participants, and provide insights for policymakers interested in applying for new waivers.^8,10^

Critically, there is much heterogeneity in the existing waiver proposals: which foods are restricted, how restrictions are defined, their motivations, evaluation designs and targeted health outcomes. This study analyzes state waiver proposals to help researchers and practitioners better understand this diversity and develop expectations of the upcoming changes to SNAP.

## Methods

States’ waiver applications [No waiver proposal was available for Louisiana. Instead, an executive order from the state’s governor (dated April 30, 2025) directing state agencies to prepare a waiver request was used to extract relevant information.] to USDA and FNS approval letters were downloaded from the agency's public website.^11^ Waiver applications follow a standard template, and include details about the rationale for the request, definitions of key terms, implementation procedures, and plans for evaluation. To systematically capture key data from the documents, an extraction guide was developed. This included data fields for the dates of application submission and approval, implementation date, requested waiver duration, restricted items (food or beverage) and associated definitions and exemptions, justifications for restriction, evaluation design and outcomes to be studied, and other state-administered programs implicated in the restriction or its evaluation (eg, SNAP-Ed, Summer EBT, Medicaid). Framework analysis was iteratively applied to the resulting dataset to describe the prevalence of different characteristics across states, and to identify prevalent themes and outliers.^12^ Administrative data were used to further contextualize the number of stores and participants affected in each state.^1,13^ The policy database and extraction sheet are available as Supplementary Data.

## Results

There were over 10.3 million SNAP participants in the 12 states with approved waivers, accounting for 24.8% of participants nationwide. A similar proportion (25.1%) of all SNAP-approved retailers will be affected by restriction policies in waiver states. The average estimated rate of SNAP participation (ie, participating population divided by total eligible population) among waiver states (86.5%) was comparable with the national average (88%), with a large range (59% in Arkansas, 100% in Colorado). On average, waivers were approved 47 days after submission to USDA (range 25-83 days). Nine states will begin implementing restrictions in January 2026 (FL, IA, ID, IN, LA, NE, OK, UT, WV), with 2 implementing in the spring (CO and TX), and the last (AR) in July 2026. Idaho takes the most staggered implementation approach, with an initial period of piloting and feedback, followed by statewide implementation by December 2026. Texas and West Virginia afford a post-implementation period for retailers to come into compliance (9 and 12 months, respectively), though most state proposals do not include such provisions. Table 1 provides a high-level view of approved waiver characteristics.

**Table 1.** Overview of approved state waiver requests for SNAP restriction.

	Date submitted	Date approved	Days to approval	Waiver start	SNAP participants^a^	Restriction
**Arkansas**	4/15/25	6/10/25	56	7/1/26	239 748	Soda, fruit and vegetable drinks (<50% natural juice), unhealthy drinks, candy
**Colorado**	5/13/25	8/4/25	83	3/1/26	614 843	Soft drinks
**Florida**	5/29/25	8/4/25	67	1/1/26	2 943 012	Soda, energy drinks, candy, prepared desserts
**Iowa**	4/1/25	5/22/25	51	1/1/26	267 158	All taxable food items^b^
**Idaho**	5/16/25	6/10/25	25	1/1/26^c^	133 545	Soda, candy
**Indiana**	4/15/25	5/22/25	37	1/1/26	586 403	Soft drinks, candy
**Louisiana**	6/27/25	8/4/25	38	1/15/26	803 988	Soft drinks, energy drinks, candy
**Nebraska**	4/14/25	5/19/25	35	1/1/26	150 600	Soda, energy drinks
**Oklahoma**	6/10/25	8/4/25	55	1/1/26	692 477	Soft drinks, candy
**Texas**	6/26/25	8/4/25	39	4/1/26^d^	3 455 085	Sweetened drinks, candy
**Utah**	5/16/25	6/10/25	25	1/1/26	177 087	Soft drinks
**West Virginia**	6/10/25	8/4/25	55	1/1/26^e^	273 566	Soda

Abbreviation: SNAP, Supplemental Nutrition Assistance Program.

^a^As of May 2025 (KFF Data).

^b^As defined by the Iowa Department of Revenue, except food producing plants and seeds for food producing plants.

^c^Phased implementation; Full implementation by December 31, 2026.

^d^Retailers have 9 months after implementation to comply.

^e^Retailers have 12 months after waiver approval to comply.

### Items restricted

Some kind of beverage restriction was proposed in all state waivers. Waivers focused on descriptions of soda (*n* = 4), soft drinks (*n* = 7), with most including the use of artificial sweeteners (*n* = 10). “Energy drinks” were also included in restrictions for 4 states. About half of waivers specifically referenced sugar (*n* = 5), with 2 states (FL, TX) using a threshold amount of 5 g to define restricted products. Added caffeine was also cited in 4 states, with 1 state (FL) using a threshold of 65 mg per 8 oz to define restricted goods. Common exemptions to beverage restrictions included products containing >50% fruit or vegetable juice by volume (*n* = 9), milk products (*n* = 8) or milk substitutes (*n* = 7). Sports drinks (*n* = 2) and coffee or tea products (*n* = 2) were also exempted in some states.

Eight states waivers proposed restrictions on candy, though these foods are variously defined. Common descriptors include mention of artificial sweeteners (*n* = 7), sugar (*n* = 6), and flour (*n* = 4), though no specific nutrient thresholds were given. Prepared desserts were also restricted in Florida, while many baked goods (regardless of nutritional content) were exempted from restriction in Oklahoma due to conflict with existing state law. Other specific exemptions are provided for granola bars (*n* = 2) or protein bars (*n* = 1), as well as items requiring refrigeration (*n* = 2), and products used for baking (*n* = 2).

Four states cite established food item definitions from industry (AR) or state code (CO, IA, TX). Table 2 describes the beverage and food restrictions by state, including key ingredients, descriptors, and exemptions used in waiver requests.

**Table 2.** Beverage and food items restricted under approved SNAP waivers, by state.

Item type	Descriptions	Detail	States												
			AR^a^	CO^b^	FL	IA^c^	ID	IN	LA	NE	OK	TX^d^	UT	WV	Total
Beverages	Main terms	Soft drinks		X		X		X	X	X	X		X		7
		Soda	X		X		X			X				X	5
		Energy drinks			X				X	X	X				4
		Sports drinks									X				1
		Low/no-calorie soda	X												1
		Unhealthy drinks	X												1
	Key ingredients and descriptors	Carbonated			X	X			X	X	X		X	X	7
		Non-carbonated				X			X^e^	X^e^	X^f^				**4**
		Fruit and veg drinks (<50% juice)	X		X	X			X		X		X		6
		Sweetened teas									X				1
		Flavored water with added sweeteners				X					X				2
		Mixes/concentrates of restricted items				X									1
		Sweeteners					X		X	X	X				4
		Natural sweeteners		X		X		X			X				4
		Artificial sweeteners		X	X	X		X	X	X	X	X	X	X	10
		High fructose corn syrup							X	X				X	3
		Sugar			X^g^					X		X^g^	X	X	5
		Caffeine			X^h^				X	X				X	4
		Advertising			X						X				2
	Exemptions	Flavored water	X		X				X					X	4
		Milk products		X			X	X	X		X	X	X	X	8
		Milk substitutes		X			X	X	X		X	X	X		7
		Sports drinks	X							X					2
		Coffee or tea			X						X				2
		Sweetened only with juice						X							1
		Fruit and veg drinks (>50% juice)		X	X		X		X		X	X	X		7
		Fruit and veg juice												X	1
		Mixes/concentrates					X								1
		Medically necessary products								X					1
Foods	Main terms	Candy	X		X	X	X	X	X		X	X			8
		Prepared desserts			X										1
		Confectionary products	X												1
	Key ingredients and descriptors	Sugar			X	X	X	X	X		X				6
		Honey				X	X	X	X						4
		Natural sweeteners				X		X	X		X	X			5
		Artificial sweeteners	X		X	X		X	X		X	X			7
		Flour	X			X			X		X				4
		Chocolate			X	X	X	X	X		X	X			7
		Bars, drops, pieces			X	X	X	X	X			X			6
		Gum				X					X	X			3
		Caramels			X	X					X				3
		Gummies			X	X					X				3
		Mints and breath mints				X					X				2
		Candy-coated fruit				X						X			2
		Taffy				X					X				2
		Licorice				X					X				2
		Jelly beans				X									1
		Candy decorations				X									1
		Sweetened fruit leathers				X									1
		Marshmallows				X									1
		Sweetened coconut				X									1
		Granola bars				X									1
		Candy-coated popcorn				X									1
		Sweetened nuts										X			1
		Nut mix with more than incidental candy				X									1
		Advertising									X				1
	Exemptions	Baking ingredients							X			X			2
		Refrigeration					X	X							2
		Granola bars				X^i^			X						2
		Protein bars							X						1
		Baked goods									X				1

Abbreviation: SNAP, Supplemental Nutrition Assistance Program.

^a^Cites item definitions from GS1 US data quality framework.

^b^Cites item definitions from §39-26-707, C.R.S.

^c^Cites item definitions from Chapter 423 of the Code of Iowa and Iowa Administrative Code chapter 701-220.

^d^Cites item definitions from Texas Administrative Code, Chapter 34, Section 3.293 (a)(4).

^e^Energy drinks.

^f^Sweetened beverages, sports drinks.

^g^>5 g.

^h^65 mg/8 oz.

^i^With flour.

### Proposed evaluations

Details of planned evaluations were provided by states in their waiver applications and referenced in USDA approval letters, and varied widely in their level of detail (see Table S1). Evaluations were most commonly described as “mixed methods” (only indicating the use of both quantitative and qualitative data) (*n* = 7), and/or included the use of a pre/post analysis (*n* = 7). Research designs also included longitudinal data analysis (*n* = 4), the use of comparison groups (*n* = 2), and difference-in-difference analysis (*n* = 1). Several states proposed evaluations within the context of SNAP-Ed (*n* = 4), and 3 proposals include children in some part of the analysis. As data sources, states hoped to use retailer data (*n* = 12), SNAP participant surveys (*n* = 11), dietary recalls (*n* = 4), Medicaid claims data (*n* = 4), food diaries (*n* = 1), interviews (*n* = 1), and data from an existing cohort study (*n* = 1). The most common outcomes for evaluation included purchasing patterns (*n* = 10), with fewer states studying dietary changes (*n* = 5) and biometric or disease outcomes (*n* = 5). A variety of ancillary outcomes were proposed in several states, including changes in the availability of SNAP food retailers (ID), experiences with stigma (WV), and impacts on household food budgets (IN). Four states reference retailer surveys (IN, OK, TX, WV) or interviews (WV) as part of their evaluations. Evaluation partners, including academic researchers, are explicitly mentioned in 7 proposals.

### Justifications for waiver requests

To justify their waver requests, states commonly cited a desire to align SNAP with its “intended purpose” for nutrition (*n* = 9), reduce Medicaid or healthcare costs (*n* = 8), encourage healthier eating (*n* = 8), and provide better stewardship of taxpayer dollars (*n* = 7). States also referenced SNAP spending on unhealthy foods (*n* = 6), and asserted that SNAP encouraged unhealthy eating (*n* = 4) (a claim that is contested in the research literature^14^). Concerns over health disparities (*n* = 4) and consumption of added sugars (*n* = 3) were also raised. A variety of health conditions were mentioned as concerns, especially obesity and type II diabetes (*n* = 11), as well as heart disease (*n* = 7). Some states also indicated how restrictions may support or augment other state programming, like the Farm-to-School Program in Arkansas and a “Double-Up Program” to incentivize healthy SNAP purchases in Louisiana. Besides the initiative proposed in Louisiana, no other states described complementary incentive programs. Table 3 illustrates the breadth of waiver justifications used by states.

**Table 3.** Justification for SNAP waiver requests, by state.

Themes	Description	States												
		AR	CO	FL	IA	ID	IN	LA	NE	OK	TX	UT	WV	Total
Motivations	Meet intended purpose of SNAP for nutrition			X	X	X	X		X	X	X	X	X	9
	Reduce Medicaid/healthcare costs	X		X			X	X	X	X	X		X	8
	Encourage healthier eating		X		X	X		X	X		X	X	X	8
	Better stewardship of taxpayer dollars			X		X	X	X	X		X		X	7
	Too much spending on unhealthy foods	X		X			X		X	X	X			6
	Concerns over child health	X		X				X	X				X	5
	SNAP encourages unhealthy eating	X			X		X		X					4
	Promote health		X	X						X	X			4
	Concerns over health disparities	X				X	X			X				4
	Concerns over added sugars			X		X					X			3
Health conditions or diseases referenced	Type II Diabetes	X		X	X	X	X	X	X	X	X	X	X	11
	Obesity	X		X	X	X	X	X	X	X	X	X	X	11
	Heart disease	X			X		X		X		X	X	X	7
	Hypertension	X		X					X		X			4
	Cancer						X		X		X			3
	Tooth decay, cavities			X	X							X		3
	Kidney diseases				X							X		2
	Non-alcoholic liver disease				X							X		2
	Stroke						X				X			2
	Gout				X							X		2
Other implicated programs	SNAP-Ed	X		X	X	X			X		X	X	X	8
	Medicaid				X	X							X	3
	Summer EBT		X											1
	WIC				X									1
	Farm-to-School Program	X												1
	SNAP Outreach		X											1
	Double-Up Program							X						1

Abbreviation: SNAP, Supplemental Nutrition Assistance Program.

## Discussion

For the first time in recent history, states will begin restricting the foods purchased by SNAP participants in January 2026. Beverages, mostly commonly sodas and sugar-sweetened beverages, are part of all waiver proposals, with some also taking aim at caffeinated energy drinks. Restrictions on foods—namely, candy—are more varied in their definitions and coverage, with states like Iowa giving long lists of restricted items, and other, like Arkansas, providing few details. All states include some kind of exemption to their food or beverage restrictions, though these range in scope and probable impact on participants’ ability to use SNAP benefits on similar unhealthy products. While all states have indicated a goal of universal restriction adoption (eg, all SNAP participants and retailers), achieving these changes at scale and ensuring compliance may present implementation issues, especially in states with more complex restriction and exemption approaches.

Though still provisional and subject to change, proposed state evaluations vary widely in their design, outcome measures, and data sources, even for states restricting similar items, which introduces sources of heterogeneity for future evidence synthesis. Differences in how restricted products are defined may also yield important challenges for evaluation. Additionally, key evaluation outcomes are not always well matched to the motivations for restriction. For instance, of the states citing concerns over child health (AR, FL, LA, NE, WV), only 2 (NE, WV) proposed evaluations with a child component. No states citing health disparities as justification (AR, ID, IN, OK) specifically reference these in evaluations. Eight states cited Medicaid or healthcare spending as a justification for restriction, but of these, only 2 (FL, WV) propose evaluations using Medicaid claims data. At least in these preliminary documents, few states explicitly define metrics for program success.

Four states name their SNAP-Ed programs and/or program staff as key actors in their waiver evaluation plans, and additional states reference SNAP-Ed as a mechanism for spreading awareness of restrictions among participants; these initiatives were defunded under the recent federal spending bill, along with several Farm to School initiatives, possibly reducing state capacity to implement their proposed plans for restriction or evaluation.^15,16^ Additionally, simultaneous changes to the broader social safety net, including reductions to Medicaid and SNAP benefits and the imposition of stricter eligibility requirements, may pose challenges to estimating generalizable effects of restriction, in addition to the heterogeneity of state restriction approaches.^17^ Future assessment of state evaluations as implemented (vs proposed) could more accurately describe the degree of mismatch between restriction motivations and outcomes, as well as the evaluations’ level of empirical rigor. Required quarterly reports to FNS may also provide more precise state plans for evaluations and help identify common issues in implementation.

## Conclusion

Once implemented, SNAP restrictions will enable public health researchers to empirically test for impacts on participants’ diet, health and wellbeing, which have long been debated. This study provides describes the current policy landscape to help set expectations for the impact of SNAP restrictions and their evaluations. There is a significant opportunity for policy researchers to help assess the implementation and impact of these policies across the US, and to inform researchers and policymakers on their intended and unintended effects.

## Supplementary Material

qxaf221_Supplementary_Data

## Data Availability

Data extraction sheets are available online: https://doi.org/10.7910/DVN/OX8JBV.
