# Supplementary material for: Characteristics of state waivers to establish nutritional restrictions in the supplemental nutrition assistance program
Source: Health Aff Sch. 2025 Nov 19;3(12):qxaf221. doi: 10.1093/haschl/qxaf221 (PMC12684384; doi:10.1093/haschl/qxaf221)
Supplement: qxaf221_Supplementary_Data [file qxaf221_supplementary_data.zip › Supplemental Tables.docx]

**Supplemental Table 1**. Characteristics of proposed SNAP waiver evaluations, by state

|  | **AR** | **CO** | **FL** | **IA** | **ID** | **IN** | **LA** | **NE** | **OK** | **TX** | **UT** | **WV** | **Total** | |  |
| --- | --- | --- | --- | --- | --- | --- | --- | --- | --- | --- | --- | --- | --- | --- | --- |
| **Design** | | | | | | | | | | | | | |  | |
| Mixed methods ^a^ |  |  | X | X |  |  | X | X |  | X | X | X | 7 | |  |
| Pre/Post |  | X |  | X | X | X |  | X |  | X | X |  | 7 | |  |
| Longitudinal analysis | X |  | X |  |  |  |  |  | X |  |  | X | 4 | |  |
| Comparison group |  |  |  | X |  |  |  |  |  |  |  | X | 2 | |  |
| Difference-in-difference analysis | X |  |  |  |  |  |  |  |  |  |  |  | 1 | |  |
| Convenience sampling |  | X |  |  |  |  |  |  |  |  |  |  | 1 | |  |
| Random sampling |  |  |  |  |  | X |  |  |  |  |  |  | 1 | |  |
| **Population** | | | | | | | | | | | | | |  | |
| SNAP-Ed participants |  |  |  | X | X |  |  | X |  |  | X |  | 4 | |  |
| Children |  |  |  | X |  |  |  | X |  |  |  | X | 3 | |  |
| WIC participants |  |  |  | X |  |  |  |  |  |  |  |  | 1 | |  |
| SNAP non-participants |  |  |  |  |  |  |  |  | X |  |  |  | 1 | |  |
| SNAP retailers |  |  |  |  |  | X |  |  | X | X |  | X | 4 | |  |
| **Data Source** | | | | | | | | | | | | | |  | |
| Retailer data | X | X | X | X | X | X | X | X | X | X | X | X | 12 | |  |
| Surveys | X | X | X | X |  | X | X | X | X | X | X | X | 11 | |  |
| Dietary recall | X |  |  | X |  | X |  |  |  |  |  | X | 4 | |  |
| Medicaid claims |  |  | X | X | X |  |  |  |  |  |  | X | 4 | |  |
| Food diaries |  |  |  |  |  |  | X | X |  |  |  |  | 2 | |  |
| Interviews | X |  |  |  |  |  |  |  |  |  |  |  | 1 | |  |
| Existing cohort study |  |  |  |  |  |  |  |  |  |  |  | X | 1 | |  |
| **Outcomes** | | | | | | | | | | | | | |  | |
| *Food purchasing and consumption* | | | | | | | | | | | | | |  | |
| Purchasing patterns | X | X | X |  | X | X |  | X | X | X | X |  | 9 | |  |
| Purchases of restricted items |  | X |  |  | X |  | X | X |  |  | X |  | 5 | |  |
| Out-of-state and online transactions |  |  | X |  | X |  |  |  |  | X |  | X | 4 | |  |
| Purchasing of nutrient-dense items |  |  | X |  |  |  |  |  |  | X |  |  | 2 | |  |
| Cash spending on restricted items |  |  | X |  |  |  |  |  |  | X |  |  | 2 | |  |
| Non-SNAP purchasing |  |  |  |  |  |  |  |  |  | X |  |  | 1 | |  |
| Changes in where/how SNAP is spent |  |  |  |  | X |  |  |  |  |  |  |  | 1 | |  |
| Dietary patterns | X | X |  | X |  |  |  |  | X |  | X |  | 5 | |  |
| Consumption of high-sugar products |  |  |  |  | X |  |  |  |  |  |  |  | 1 | |  |
| *Health outcomes* | | | | | | | | | | | | | |  | |
| Type II diabetes |  |  | X |  | X |  |  |  |  |  |  | X | 3 | |  |
| Obesity |  |  | X |  | X |  |  | X |  |  |  | X | 4 | |  |
| Cardiovascular disease |  |  | X |  | X |  |  |  |  |  |  | X | 3 | |  |
| Body Mass Index |  |  |  | X |  |  |  |  |  |  |  |  | 1 | |  |
| *Other outcomes* | | | | | | | | | | | | | |  | |
| Perceptions of food access |  |  |  |  | X | X |  |  |  |  |  | X | 3 | |  |
| Awareness of restriction |  |  | X |  |  | X |  |  |  | X |  |  | 3 | |  |
| Economic status of SNAP households |  |  |  |  |  | X |  |  |  |  |  | X | 2 | |  |
| Nutritional status of SNAP households |  |  |  |  |  | X |  |  |  |  |  | X | 2 | |  |
| Perceptions of healthy foods |  |  |  |  |  |  |  |  | X | X |  |  | 2 | |  |
| Impacts on food budget |  |  |  |  |  | X |  |  |  |  |  |  | 1 | |  |
| Fairness to different SNAP households ^b^ |  |  |  |  |  | X |  |  |  |  |  |  | 1 | |  |
| Experiences of stigma |  |  |  |  |  |  |  |  |  |  |  | X | 1 | |  |
| Availability of SNAP-approved retailers |  |  |  |  | X |  |  |  |  |  |  |  | 1 | |  |
| Unexpected impacts |  |  |  |  |  |  |  |  |  | X |  |  | 1 | |  |
| **Evaluation partner(s)** ^c^ | X | X | X ^d^ | X | X |  |  |  |  | X | X | X | 7 | |  |

^a^ “Mixed methods” refers to qualitative and quantitative data collection, rather than a traditional mixed methods research design, where quantitative and qualitative data are merged and synthesized at one or multiple points in an analysis.

^b^ Fairness between households of different income levels, age compositions, sizes, and regions

^c^ Indicates states that specify an external group to undertake or support evaluation efforts

^d^ Possibility of external evaluation partner referenced
